# Socio-Economic and Political Challenges of EU Member Countries: Grasping the Policy Direction of the European Semester

**DOI:** 10.1057/s41294-021-00171-2

**Published:** 2021-10-11

**Authors:** Sara Casagrande, Bruno Dallago

**Affiliations:** Department of Economics and Management, University of Trento via Inama, 5, 38122 Trento, Italy

**Keywords:** Benchmark, Content analysis, Country-specific recommendations, Distance to frontier score methodology, European Monetary Union, European Semester, Fiscal policy coordination, Policy direction, C43, C80, E61, F15, O52

## Abstract

The European Semester (ES) and the country-specific recommendations (CSRs) have been introduced with the purpose to promote flexibility and adaptation to national circumstances in the governance of fiscal policies. To assess whether the ES has contributed to reconcile economic and social objectives, we measured, through the distance to frontier (DTF) score methodology, the distance of each member country from a benchmark based on EU aims and values defined in the EU treaties. Results show that EU member countries are far from the benchmark and CSRs have not prevented a progressive deterioration of stability and cohesion from an economic, political and social perspective. A content analysis of the CSRs issued from 2011 to 2018 and a comparison with the DTF scores reveal a weak connection between member countries’ performance and CSRs. Despite the social content of many CSRs, we actually observe a “commodification” of their goals. CSRs promote a society functional to flexible and competitive markets, and compatible with the requirements of fiscal discipline and sustainability. This neoliberal approach apparently played a role in the EU deterioration and makes the “socialization” of the ES a process with ambiguous implications for European citizens.

**Supplementary Information:**

The online version contains supplementary material available at 10.1057/s41294-021-00171-2.

## Introduction

The European debt crisis has shown the fragility of the European Monetary Union (EMU) architecture, characterized by a centralized monetary policy in the face of fiscal policies managed by member countries with idiosyncratic institutions. The introduction of the European Semester (ES) in 2011 represented a response to the need to improve coordination, surveillance and reconcile economic and social objectives. Country-specific recommendations (CSRs) are the most important output of this annual economic policy coordination cycle, that seeks to overcome a one size fits all approach, to face institutional variety and to promote socio-economic integration.

Despite the undoubted merits of this new tool in promoting greater flexibility within the EU, the ES has been subject to extensive debates in literature, going from its democratic legitimacy to the alleged pre-eminence of economic objectives at the expense of social ones, up to the effectiveness of CSRs. Some scholars questioned the clarity, transparency and coherency of CSRs. Unlike many contributions that have tried to establish whether the ES has achieved its objectives or to classify CSRs according to European Commission policy areas, this paper aims to assess whether the ES has contributed to reconcile economic and social objectives, how European institutional variety and challenges have been faced, and if member countries have been induced to improve the working of their institutions and promote social progress and the well-being of citizens. To do so, we measured the distance of each member country from a benchmark based on EU aims, values and socio-economic goals defined in the EU treaties, by considering jointly and equally important the economic, political and social dimensions. Then, we compared these results with CSRs issued from 2011 to 2018 in order to evaluate whether and how the ES has promoted member countries’ socio-economic and political progress and to grasp ES policy direction.

Results show that EU member countries are far from the benchmark and CSRs have not prevented a progressive deterioration of stability and cohesion from an economic, political and social perspective. A content analysis of the CSRs and a comparison with the DTF scores reveal a weak connection between member countries’ performance and CSRs. Despite the social content of many CSRs, we observe a “commodification” of their goals. CSRs promote a society functional to flexible and competitive markets, and compatible with the requirements of fiscal discipline and sustainability. Many issues related to social conflicts, integration and equity are not really addressed. This neoliberal approach, far from the aims and values of European treatises, may have played a role in the deterioration of European integration with ambiguous implications for European citizens.

This paper proceeds as follows. After an overview of the ES structure, its economic rationale, and major issues and debates in Sect. 2, the benchmark and the distance to frontier (DTF) score methodology are described in Sect. 3. In Sect. 4, an empirical investigation of this benchmark is exposed, with an analysis of DTF scores and a comparison with CSRs. Section 5 concludes.

## European Semester: Aims, Processes and Outputs

### Economic Rationale and Processes Within the European Semester

The asymmetric structure of EMU implied that while monetary policy has started to be managed centrally by a supranational central banking system, the Eurosystem; European member countries have kept national sovereignty for fiscal policy. According to Howarth and Verdun (2020, p. 288), it was widely thought that there would be plenty of time to overcome this asymmetrical structure or strengthen an EMU clearly characterized by a “fair-weather design”. Moreover, many scholars supported the application of endogenous Optimal Currency Area (OCA) theory to the European project (e.g. Frankel and Rose 1998) and were confident that monetary integration would have induced economic and political convergence, despite the heterogeneity of European member countries’ institutional frameworks (see Dyson and Maes 2016). Unfortunately, with the outbreak of the sovereign debt crisis, “a second asymmetry emerged, one roughly between the Euro Area core and the periphery”. This asymmetry made even more evident the difficulty of bringing together, within a monetary union, member countries characterized by extremely different growth models (Hancké 2012; Hassel 2014), for which institutional convergence has proved to be more challenging than economic convergence (Schönfelder and Wagner 2019; Alesina et al. 2017). Perhaps even worse, the crisis accelerated the divergence between resilient and vulnerable countries within the Eurozone, which made the implementation and governance of common policies increasingly difficult and in the end unsustainable (Dallago 2016).

In such an emergency, the introduction of new tools capable of improving coordination, surveillance and economic convergence in a more effective way with respect to the Broad Economic Policy Guidelines (BEPGs) and the Employment Guidelines (EGs) was needed. For this reason, an annual socio-economic policy coordination cycle, the European Semester (ES), has been introduced in 2011. A first reference to the ES can be traced back to the Commission communication on *Reinforcing economic policy coordination* dated 12 May 2010. The document proposed “the establishment of a European Semester for economic policy coordination, so that Member States would benefit from early coordination at European level as they prepare their national stability and convergence programmes including their national budgets and national reform programmes”.

Surely ES economic rationale is not limited to surveillance, but it is also aiming to foster structural reforms for overcoming European institutional heterogeneity. Indeed, the purpose of the ES is also to “ensure sound public finances, prevent and correct excessive macroeconomic imbalances, foster structural reforms and boost jobs, growth and investment” (Efstathiou and Wolff 2018, p. 2). Some scholars sustain that the objective of the ES is “to transform BEPGs and EGs into a binding process” (Bénassy-Quéré 2015, p. 7) so that “EU member states align their budgetary and economic policies with commonly agreed objectives” (Haas et al. 2020, p. 331).

ES embraces within a single annual policy different legal bases and sanctioning authority such as the Stability and Growth Pact (SGP), the Macroeconomic Imbalances Procedure (MIP), the Fiscal Treaty and the Europe 2020 Strategy (Zeitlin and Vanhercke 2015, p. 65). Only the recommendations made in the context of the Excessive Deficit Procedure (EDP) and the Excessive Imbalance Procedure (EIP) are binding (Darvas and Leandro 2015, p. 4). According to Delors et al. (2011, p. 2), the ES introduced two novelties: the synchronization of different procedures and an ex-ante coordination. Indeed, before the crisis, structural reforms and budgetary surveillance followed different timing and procedures within an ex-post coordination unable to create synergies. Despite the single monetary policy remaining the main argument to coordinating budgetary policies (Alcidi and Gros 2017, p. 1), the ES connection with different sanctioning regimes makes this tool flexible enough to balance the need to limit the negative spillovers that may arise within a monetary union with the degree of interference in national policies (Haas et al. 2020).

The ES cycle lasts 6 month and starts in November with the autumn package provided by the European Commission (EC). It includes the annual growth survey, the most important tool for defining socio-economic priorities, and the alert mechanism report, which may be followed by an in-depth review and represents the base for the annual macroeconomic imbalance procedure (MIP). After this preparatory phase, in January, the Council of the EU discusses the annual growth survey and provides policy guidelines. In March, the EC publishes country reports which may include in-depth reviews of macroeconomic imbalances. In the next phase, member countries submit their policy plans (stability and convergence programmes and national reform programmes) taking into account European policy guidelines. The EC evaluates policy plans and presents accordingly draft country-specific recommendations (CSRs), which will be discussed and approved by the Council of the EU. In the next months, it is expected that member countries will apply the CSRs received. Although tailor-made recommendations are not a novelty introduced by the ES (see Zeitlin 2010), it is out of doubt that CSRs played a new key role within the ES and represent its most important output. Country reports and the CSRs are the result of the analysis of different scoreboards and dialogues with member countries. Various scoreboards have been added over the years as a basis for analysis to develop CSRs: from the MIP scoreboard introduced in 2011, the EU Justice Scoreboard introduced in 2013, to the social scoreboard introduced in 2017 and that accompanies the European Pillar of Social Rights with the aim of monitoring the social performance of member countries.

The evolution of scoreboards seems to testify the reorientation of the ES towards a more socially balanced policy, with a stronger revaluation of social objectives (see Zeitlin and Vanhercke 2018). As explained by Clauwaert (2013, p. 5), from a social point of view, the purpose of CSRs is to pursue the Europe 2020 strategy, a successor of the Lisbon strategy, that aims to “deliver more growth that is simultaneously ‘smart’ (by investing more in education, research and innovation), ‘sustainable’ (by, among other things, moving in the direction of a low-carbon economy) and ‘inclusive’ (by boosting job creation and reducing poverty)” for the 2010–2020 decade. In 2016, the EC presented the EU 2030 Agenda, based on Sustainable Development Goals (SDGs) for fostering sustainable development. As part of the Green Deal, the EC promoted in his 2020 work programme the integration of the SDGs into the ES. Progress towards the SDGs has been taken into account into 2020 country reports.

Different policy areas are considered within the ES. These policy areas have changed in time, coherently with the inclusion of new scoreboards and now the EC considers 32 policy areas. It is possible to assign each recommendation to one or several policy areas, but, as confirmed by Efstathiou and Wolff (2019, p. 3), this matching procedure, carried out by the EC, is not made public. Apart from the MIP scoreboard, in which violations of the thresholds are quite evident, it is not clear how scoreboards influence the final CSRs, which remain the result of a complex evaluation process. As confirmed by Costello (2017, p. 1), “the recommendations are based on the findings of the Country Reports and on the dialogue with Member States, national parliaments, social partners, civil society and other stakeholders”. This may indicate the intention of the European institutions to overcome a “logic of thresholds” and to issue CSRs starting from a dialogue with the social parts involved. However, this makes a true understanding of CSRs and European strategies more challenging.

### European Semester: Progresses, Issues and Challenges

According to Zeitlin and Vanhercke (2015, p. 65), the ES “has given EU institutions a more visible and intrusive role in scrutinizing and guiding national economic, fiscal and social policies”. In this regard, Delivorias and Scheinert (2019, p. 2) note how “while [ES] involves no legal transfer of sovereignty from the Member States to the EU level, it has given the EU institutions a more visible and, in some respects, also more authoritative role than ever before in monitoring, scrutinizing and guiding national economic, fiscal and social policies, especially within the euro area”. However, Verdun and Zeitlin (2018, p. 144) note how “Member states do not control the European Semester, nor have supranational institutions become all-powerful”, indeed “to enhance national ownership of proposed reforms, the Commission now engages in an increasingly intense bilateral and multilateral dialogue with a multiplicity of actors from the member states at each stage in the Semester cycle” (p. 145).

The introduction of the ES seems to have somehow replaced the *one size fits all* approach, indeed “the recommendations that the different Euro Area members have received over time vary according to country, year and economic model” (D’Erman et al. 2019, p. 206). The ES has changed significantly over time with the aim of incorporating social objectives. Many scholars recognize a progressive socialization of the ES confirmed by the social content of many CSRs (Urquijo 2017; Zeitlin and Vanhercke 2015). Verdun and Zeitlin (2018, p. 144) claim that “social objectives and actors have become more prominent and influential over time in the Semester’s substantive policy orientations and governance procedures, including the drafting, review and adoption of the CSRs”.

However, “the rapid evolution of the European Semester has raised a series of hotly contested theoretical, empirical and normative questions” (Zeitlin and Vanhercke 2015, p. 66). Among the most debated topics, it is possible to find the alleged subordination of social objectives to fiscal sustainability requirements. According to some scholars, economic objectives remain a priority (Parker and Pye 2018). Indeed, while social objectives are subject to weak forms of surveillance and enforcement, on the basis of the voluntary Open Method of Coordination (OMC), macroeconomic objectives are pursued through strong surveillance and binding correction mechanisms such as the MIP (De la Porte and Heins 2015). This imbalance between economic and social objectives is probably the basis of the proposal to introduce a “Social Imbalances Procedure” (Sabato et al. 2019). Given that many social objectives are in stark contrast to the constraints of fiscal discipline, social goals and policies are considered by some scholars destined to go into the background (Hyman 2015; Graziano and Hartlapp 2019). Copeland and Daly (2018, p. 1002) sustain that “the degree of progress towards social policy in the European Semester (2011–15) has been conditional and contingent” and that “EU social policy as enunciated through the CSRs is much more oriented to supporting market development than it is to correcting for market failures”. The role of the social dimension within the EMU is a highly debated issue that goes beyond the ES and deals with the structure of the EU itself (see Haas et al. 2020; Minkkinen and Patomäki 1997). The way in which the crisis was managed led many scholars to believe that “EU social policy seems to be characterized by a tension of high hopes and limited realization” (Graziano and Hartlapp 2019, p. 1485). Another point of discussion concerns the effect of the ES on relationship between European institutions and member countries and on the level of democracy of the decision-making process (see Verdun and Zeitlin 2018; Crum 2018).

Finally, discussions were held on the effectiveness of the ES in inducing national reforms. This seems to put into discussion the effectiveness of the attempts made to improve surveillance and the authoritative role of the ES. This topic was addressed, for example, by analysing the degree of implementation of CSRs that EC evaluates through 3 categories: no progress, some progress, substantial progress. As commented by Efstathiou and Wolff (2019, p. 4) “in aggregate, from 2013 to 2018 member states made ‘limited progress’ or ‘some progress’ with the recommendations they received as part of the European Semester […] Worse, implementation of recommendations worsened over time”. The authors recognize the tendency of policymakers to implement reforms when the time is politically right, or the risks are high. Similarly, Darvas and Leandro (2015, p. 19) claim that ES “has been rather ineffective” also because national policymakers pursue national interest that “differ widely in different member states”. However, Efstathiou and Wolff (2018, p. 14) underline the importance of developing good recommendations, indeed “the current form of CSRs makes for barely digestible documents. More streamlined and understandable communication would be useful”. Haas et al. (2020, p. 338) comment that CSRs “tend to recommend reducing public spending; they also encourage more social protection for vulnerable groups. Given the tension inherent in combining such recommendations, the Semester’s limited implementation record may appear less surprising”.

The alleged lack of clarity and transparency in CSRs and MIP is a recurring topic. For example, it was pointed out that the “classification of Member States with imbalances lacks transparency, the Commission’s in-depth analysis despite being of a good standard has become less visible and there is lack of public awareness of the procedure and its implications” (European Court of Auditors 2018, p. 85). Although the economic and social indicators and databases used within the MIP are public, it is not clear how they influence the in-depth analysis. Similarly, it is not clear how many scoreboards feed concretely into the ES while the coexistence of different scoreboards has led to an overlapping of indicators (Galgóczi et al. 2017). CSRs are not simple to be interpreted. For example, Haas et al. (2020) tried to code the policy direction of CSRs and only 64% of the CSRs contained a language that unambiguously indicates a policy direction. Moreover, the authors admitted that “while the content of CSRs is interesting in and of itself, it tells us little about the hierarchy between recommendations” (p. 333).

Doubts were raised also on the appropriateness of indicators and scoreboards. For example, with reference to the MIP scoreboard, the casual relevance of specific indicators and upper and lower thresholds has been object of debate. Many indicators refer to phenomena which are not under the direct control of national governments (Scharpf 2011). According to Biegun and Karwowski (2020), only some MIP indicators are significant for predicting crises. Bénassy-Quéré and Wolff (2020) sustain that the MIP could be simplified, and its underlying conceptual framework clarified. The authors underline how the MIP regulation is somewhat contradictory on how to deal with current account imbalances and that it remains largely a country-by-country approach, unable to quantify intra-EU imbalances with the risk of aggravating the deflationary bias in the euro area.

## Assessing EU Member Countries Progress and Challenges: Towards New Strategies

### From Treaties to Long-Term Goals: Defining a Benchmark

The lack of clarity and transparency of CSRs and the ambiguous role of scoreboards in their definition make it difficult to understand the “policy direction” of the EU’s recommendations (D’Erman et al. 2019) and in general the long-term strategies and real priorities of European institutions. Unfortunately, this seems to be a persistent issue. As noted by Renda (2017, p. 2), “when it comes to medium-term growth and development strategies, the European Union has not been very successful over the past two decades […]. Today, Europe de facto has no strategy in place for the end of this decade”.

A long-term strategy must be linked to the deep EU values and objectives that are present in the constitutive treaties of the EU. The effectiveness and adequacy of the ES should be evaluated within a theoretical framework capable of evaluating the progress made by member countries in achieving the long-term European objectives. For European objectives, we mean the aims and values of the EU that have been agreed upon by all member countries through the approval of community treaties. These aims and values should be achieved along a multi-dimensional process of integration. There are three levels of European integration: economic integration as a prerequisite for political and ultimately social integration (Eppler et al. 2016). Consequently, it is coherent with literature and European treaties to evaluate the achievement of EU objectives along 3 dimensions: economic, social and political.

EU objectives can be grouped according to some main categories. The identification of these categories can be traced to one of the most influential reports that preceded (and promoted) the EMU creation, the Padoa-Schioppa report (Padoa-Schioppa 1987). The report identified three keywords: efficiency, stability and equity. These have been considered the initial 3 pillars (Sapir et al. 2003) of the European project, which together with an “actual growth performance” should “in the Group’s judgement, be the basis of the long-term ‘social contract’ between the Community and all its Member States” (Padoa-Schioppa 1987, p. 5).

We sustain that by combining the 3 pillars and the 3 dimensions, we have the backbone of the theoretical framework to analyse the degree of achievement of the European objectives. It is useful to consider that the interpretation of the 3 pillars in all the 3 dimensions brings some changes to the pillars. The economic literature clearly shows that efficiency is often considered together with effectiveness in different fields (e.g. Aubyn et al. 2009; Mandl et al. 2008; Montes et al. 2019; Ringel and Knodt 2018). Indeed, an optimal use of resources aimed at minimizing costs cannot ignore an assessment of the quality of the results and their consistency with the objectives, and this is even more relevant within the EU context, in which not only economic but also social objectives are considered. It is worth remembering the relevance of the “principle of effectiveness” for EU law, which with other principles represents “a weak notion of virtues that have together been used as a substitute for any ‘strong’ ethical, or ideal, foundation” (Williams 2009, p. 551). The concept of socio-economic equity cannot be separated from that of political equality. Indeed, more productive agents have the right to be rewarded properly, according to the principle of the equality of citizens, as stated in the EU Charter of Fundamental Rights.

Taking into account these observations, the combination of pillars and dimensions generates 9 areas for evaluating progresses towards European objectives. These areas are: economic efficiency and effectiveness, economic stability, economic equity, social efficiency and effectiveness, social stability, social equity, political efficiency and effectiveness, political stability and political equality. To characterize each area, we use the aims and values of the EU, which can be traced firstly in the Treaty on European Union (TEU) and then in the ill-fated Treaty Establishing a Constitution for Europe (TECE). Subsequently, these principles and values (some already present in TEU) have then been repeated in the Lisbon Treaty. According to some scholars, TEU and TECE provide an expression of EU “moral identity” (Williams 2009, p. 555) despite the fact that “we are no nearer a clear understanding of what the European Union is for or the values that govern its development and practice than we were in 1957” (pp. 552–553).

We proceeded with a content analysis of the TEU and the TECE, by identifying keywords that are coherent with each area. The content analysis has been conducted with the help of a software (we started with word cloud and bag-of-words analysis using MATLAB Text Analytics Toolbox algorithms). Thanks to this analysis, we identified 19 indices for assessing EU member countries progresses towards declared EU values and aims (see Table 1).

1. *Economic efficiency and effectiveness*: according to TEU, “the Union shall establish an internal market” [art.3(3)] in which the “free movement of persons, services, goods and capital, and freedom of establishment shall be guaranteed within and by the Union” [TECE art.I-4(1)]. In all markets, EU’s aim is to guarantee the “efficient functioning of the institutions” (TEU, preamble), promote a “highly competitive social market economy” [TEU, art. 3(3)] in which dominates “free and fair trade” [TEU, art. 3(5)] and “competition is free and undistorted” [TECE, art.I-3(2)]. The objective is the achievement of “economic and social progress” and “sustainable development” (TEU, preamble). The role of “environmental protection” is relevant for EU, which clearly states the goal to guarantee a “high level of protection and improvement of the quality of the environment” [TEU, art. 3(3)]. In addition to this, EU “shall promote scientific and technological advance” [TEU, art. 3(3)]. With reference to labour markets the EU is “aiming at full employment and social progress” [TEU, art. 3(3)] with attention to the vocational training of young people (TECE, Sect. 5). Workers have the right to access to vocational, continuing training and lifelong learning, the right to improve employment or self-employment prospects, to achieve a work–life balance within a workplace contest aimed to guarantee social dialogue and the involvement of workers (see also TECE, art.III-203,204,209,210,283). Within financial markets, it is expected that the banking systems contribute to an “efficient allocation of resources” (TECE, art.II-2).
2. *Economic stability*: the union aims at achieving a “balanced economic growth and price stability” [TEU, art. 3(3)]. “Stable prices, sound public finances and monetary conditions and a stable balance of payments” are considered “guiding principles” of EU economic and monetary policy (TECE, ch.2, art. III-177). The treaty therefore refers to a long-term economic sustainability that can be obtained in conditions of stability of the financial markets [TECE, art. II-185(5)] and economic soundness. The goal of economic soundness is not limited to “raising growth potential” (e.g. through environmental and research and development policies) and “securing sound budgetary positions” (TECE, Title VII, art.17). The covid-19 emergency is fostering greater awareness of the importance of limiting the EU’s vulnerability and dependence on foreign partners. This concept is confirmed in TEU [art. 3(3)] and TECE [art. I-3(4)]: “in its relations with the wider world, the Union shall uphold and promote its values and interest” and shall promote an “harmonious development of world trade” TECE (art. III-314). It is quite obvious that this harmony should be guaranteed also within the Union. From this awareness comes the recent goal promoted by the European institutions to aim for a “strategic sovereignty for Europe” (see Anghel 2020).
3. *Economic equity*: The EU promotes “fair trade” and aims at promoting the “eradication of poverty” [TEU, art. 3(5)] and “social justice” [TEU, art. 3(3)]. Fair trade implies fairness in business with particular attention to the minority shareholders’ interests (see for example the *EU Action Plan* in 2003 and 2012 aimed to strength shareholders’ rights, limit conflict of interest, promote shareholder engagement and disclosure). The commitment for the eradication of poverty is an objective confirmed in Europe 2020 Strategy, in The European Pillar of Social Rights, and it is part of European Social Model. It is connected to the commitment to promote a more equitable distribution of income (redistributive justice as a concept connected to “social justice”), adequate working remuneration and conditions (TECE, art. III-209), social security benefits and social assistance protection to families and people “in cases such as maternity, illness, industrial accidents, dependency or old age, and in the case of loss of employment, in accordance with the rules laid down by Union law and national laws and practices” (TECE, Title IV, art. II-94).
4. *Social efficiency and effectiveness*: as confirmed by Lenzi and Zoppè (2020) “public expenditures characterize the ‘social market economy’, mentioned in the EU Treaty as one of its aims”. Three spending areas (beyond those devoted to improve equity, inclusion and reduce poverty) deserve particular attention as drivers of future EU prosperity and social progress: healthcare, education and research and infrastructure. Healthcare is certainly the area of expenditure destined to play an increasingly important role; indeed, the covid-19 crisis has shown the economic impact of public health. Healthcare is also part of the principles of The European Pillar of Social Rights and it is present in the TECE, art. II-95, which states: “a high level of human health protection shall be ensured in the definition and implementation of all Union policies and activities”. Health and education have been considered jointly in TECE (art. III-117), which claims that “in defining and implementing the policies and actions” the Union should promote “high level of education, training and protection of human health”. Education and research are at the base of “three long-awaited strategic proposals”: the *Digital Education Action Plan* (2021–2027), the *European Research Area*, and the *European Education Area* (to be achieved by 2025). According to TECE (art. III-246), infrastructure development is a prerequisite to “derive full benefit from the setting-up of an area without internal frontiers”. It also promotes “the establishment and development of trans-European networks in the areas of transport, telecommunications and energy infrastructures” and helps to European countries including regions with low infrastructure development (e.g. see TECE—Declaration concerning Italy).
5. *Social stability*: The UE aims to guarantee public “safety and security” (TEU, preamble) also by engaging in “the prevention and combating of crime” [TEU, art. 3(2)]. Close to this goal, the UE intends to prevent social conflicts by promoting “peace” [TEU, art. 3(2)], protection of the “rights of persons belonging to minorities”, “pluralism, non-discrimination, tolerance” [TEU, art. 3(2)]. In this regard, TECE claims that “the Union shall endeavour to ensure a high level of security through measures to prevent and combat crime, racism and xenophobia, and through measures for coordination and cooperation between police and judicial authorities and other competent authorities” (art. III-257(3), see also Sect. 4). Consequently, public security and the eradication of social conflict are the building blocks of European social stability.
6. *Social equity*: The EU promotes “solidarity” (“between generations” but also “among Member States” and “among peoples”), and the fight against “social exclusion and discrimination” [TEU, art. 3(3 and 5)]. In TECE it is stated that “any discrimination based on any ground such as sex, race, colour, ethnic or social origin, genetic features, language, religion or belief, political or any other opinion, membership of a national minority, property, birth, disability, age or sexual orientation shall be prohibited” [art. II-81(1)]. These aims are confirmed in the principles of The European Pillar of Social Rights. The promotion of solidarity calls into question an effort to develop a cultural attitude aimed at inclusion and legality. The importance of legality is confirmed in TECE (Sect. 4), which explicitly aims to prevent and combat corruption. Corruption, in addition to being a crime, can be considered, together with other phenomena (e.g. bribes, tax avoidance etc.), a set of factors that hamper the EU ability “to continue along the path of civilisation” and to “deepen the democratic and transparent nature of its public life” (TECE, preamble).
7. *Political efficiency and effectiveness*: The EU aims to promote the “efficient functioning of the institutions so as to enable them better to carry out, within a single institutional framework, the tasks entrusted to them” (TEU, preamble). Although the EU does not set itself the goal of affecting directly national political systems, there is no doubt that the integration process influences national political institutions (Hix and Goetz 2000) and that European institutions promote national political efficiency and effectiveness. Indeed, political efficiency and effectiveness are prerequisite for ensuring justice, equality and democracy. It is possible to classify political institutions according to the *trias politica* model, which identifies 3 branches: legislative, executive and judiciary.
8. *Political stability*: The EU aims to promote the “democratic and efficient functioning of the institutions” (TEU, preamble). In several TEU places, reference is made to the commitment to “ensure that the Union’s actions are coherent and transparent” (art.11). According to the Treaty, the “decisions shall be taken as openly and as closely as possible to the citizen”, for preserving the quality of “democratic life” [art.10(3)] and with the purpose of developing a “political awareness and to expressing the will of citizens” [art.10(4)]. Institutions should “give citizens and representative associations the opportunity to make known and publicly exchange their views” [art.11(1)]. Moreover, “the institutions shall maintain an open, transparent and regular dialogue with representative associations and civil society” [art.11(2)]. It seems reasonable to assume that these objectives should be ensured within each member country and that a “democratic society” (TECE) is a prerequisite of political stability.
9. *Political equality*: The EU ensures “rights of the human person, freedom, democracy, equality and the rule of law” (TEU, preamble) and in particular the “equality of its citizens, who shall receive equal attention from its institutions, bodies, offices and agencies” (TEU, art.9). The independence and impartiality of political institutions are prerequisite for ensuring political equality and democratic values between and within institutions.

**Table 1:** Pillars, dimensions, areas and indices

Pillars/dimensions	Economic	Social	Political
Efficiency and effectiveness	goods market; labour market; financial market	health system; education and research system; infrastructure	legislative branch; executive branch; judiciary branch
Stability	financial stability; economic soundness	public security; social conflict	political system and government stability
Equity/equality	fairness in business; economic equity	social inclusion; culture of legality	independence, impartiality and democracy

### The Distance to Frontier (DTF) Score Methodology

Efficiency, effectiveness, stability, equity and equality are recurrent themes in the European treaties and reports, and relevant for assessing European member countries performance, as explained in Casagrande and Dallago (2021a, b), who introduce the European benchmark, and use the distance to frontier (DTF) score methodology for investigating European institutional variety.

The DTF score methodology is a valid instrument also for measuring progresses and identifying problems within our benchmark, which can be interpreted as a frontier against which to measure progresses and identify issues for each EU member country through the 19 indices summarized in Table 1. These are composite indices, that can be constructed starting from the aggregation of different indicators and that will be defined in this section.

The DTF is an absolute score. It allows to compare a member country’s performance against the best and worst performance for each indicator, to measure a country’s relative position and to compare index scores over time. The indicators used in the DTF scores computation can present different units of measurement (e.g. values, percentages, quantities, years, etc.). As explained in WB (2018), indicators are firstly normalized to a common unit for meaningful comparisons. Then, each indicator y is rescaled using the linear transformation (worst–y)/(worst–frontier). Then, the scores obtained for each indicator and for each country are aggregated into one distance to frontier score. The DTF scores are indicated on a scale from 0 to 100, where 0 represents the worst performance and 100 the best potential performance, the frontier. The best and the worst performance can correspond to values which may not be achieved by any country. For example, an indicator could be represented by answers of citizens or experts to questionnaires ranging from a minimum to a maximum score set by the interviewer. The answers range within this scale without necessarily reach the minimum and/or the maximum score. In other cases, the indicator’s values may come from a technical assessment done on the government’s policy implementation. Considering that indices are the combination of different indicators, the frontier does not correspond necessarily to the performance of a particular country.

The choice of the indicators used in the DTF score computation has been conducted by resorting to the guidelines of the *Handbook on Constructing Composite Indicators* (OECD and JRC 2008), which suggests a fitness-for-purpose principle starting from the development of a theoretical framework and claims that “indicators should be selected on the basis of their analytical soundness, measurability, country coverage, relevance to the phenomenon being measured and relationship to each other” (p. 15). In our case, the benchmark is the theoretical framework from which to start a process of indicators’ selection. Indicators have been chosen according to the following indices’ definitions based on TEU and TECE content analysis:

1. *Goods market efficiency and effectiveness*: grade of competition among business and companies, the environmental sustainability of production processes, and the technological content of production.
2. *Labour market efficiency and effectiveness*: ability of the labour markets to limit unemployment, to promote workforce career prospects and lifelong learning and to guarantee social dialogue and the involvement of workers.
3. *Financial market efficiency and effectiveness*: grade of financial depth, financial competition and ability to allocate resources efficiently by meeting the needs of the real economy, including small and medium enterprises.
4. *Financial stability*: financial markets stability and banking system soundness.
5. *Economic soundness*: efficiency and effectiveness of government spending, environmental, research and development policies aimed at promoting sustainable growth, and limited dependency on foreign decisions and resources.
6. *Fairness in business*: grade of protection of investors, minority shareholders’ interests and property rights and the strength of auditing and reporting standards.
7. *Economic equity*: ability to fight income inequality, poverty risks, and to ensure social security benefits and social assistance protection.
8. *Health system efficiency and effectiveness*: availability and quality of health system structures and medical equipment, and the efficiency and affordability of healthcare services.
9. *Educational and research system efficiency and effectiveness*: enrolment rates, minimal educational qualifications, quality of results, incidence of researchers, quality of education systems and facilities.
10. *Infrastructure efficiency and effectiveness*: quality of important infrastructures.
11. *Public security*: occurrence of crime, safety perceptions and the reliability of policy services.
12. *Social conflict*: perceived tension between different social groups and the incidence of protest and demonstrations.
13. *Social inclusion*: difficulties in social inclusion also due to discrimination.
14. *Culture of legality*: incidence of corruption, tax evasion and organized crime as proxies of the social attitude towards legality and, consequently, that social ethical behaviour which is at the base of solidarity.
15. *Legislative branch efficiency and effectiveness*: government ability to take shared decisions and approve and enforce laws efficiently.
16. *Executive branch efficiency and effectiveness*: public administration efficiency and reduction of the negative impacts of bureaucracy.
17. *Judiciary branch efficiency and effectiveness*: ability of the judicial system to apply laws and resolve conflicts promptly, with rapid and fair procedures.
18. *Political system and government stability*: government stability and participation of the electorate in political life.
19. *Independence, impartiality and democracy*: independence and impartiality of political institutions, democratic values between and within institutions and society.

In the index “economic soundness”, we have not considered sovereign debt sustainability using Maastricht thresholds. Despite fears relating to possible drops in economic growth when a country’s level of government debt exceeds certain thresholds, as claimed in some controversial studies such as that of Reinhart and Rogoff (2010), many scholars have questioned Maastricht thresholds. Some scholars claim that these thresholds lack a theoretical support (Pasinetti 1998a, b) also because debt sustainability is a controversial concept (Neck and Sturm 2008). Some claim the need to reform these rules (Darvas 2010) or at least to take into account the impact on growth prospect (Zuleeg and Schneider 2015; Truger 2015). These claims seem coherent with TECE, which confirms the importance of preserving growth prospects and does not rule out discussions on the SGP. In detail, TECE claims that “raising growth potential and securing sound budgetary positions are the two pillars of the economic and fiscal policy of the Union” and that “Member States should use periods of economic recovery actively to consolidate public finances” so that to create “the necessary room to accommodate economic downturns”. Despite promoting SGP, TECE clarifies that this does “not prejudge the future debate on the Stability and Growth Pact” (TECE, Title VII, art.17). Without going into the detail of this debate, we sustain that an unsustainable level of sovereign debt must be necessarily associated to financial instability, a lack of economic soundness and a lack of efficiency and effectiveness of public spending, dimensions considered within the benchmark.

The indicators have been collected from different databases^1^ and chosen according to their soundness, measurability, country coverage, and relevance. The number of indicators varies depending on the index because their complexity is not the same. In total, 189 indicators have been selected (see Table H and I in the Supplementary Material) and have the same weight because all indices and benchmark’s areas represent objectives with equal importance for European institutions. As aggregation method, the geometric mean has been used. Geometric aggregation not only avoids that poor performance in one indicator may be compensated by a better performance in another indicator, but encourages countries to improve their weaker dimensions and penalizes those countries with unbalanced profiles. These aspects are coherent with the purposes of our analysis, which aims to underline how countries should pay equal care to all dimensions (and indices) coherently with EU goals (see in the Supplementary Material Figure E and Table F for a comparison of DTF scores using the arithmetic aggregation). Given the strong heterogeneity of the indicators, the problem of missing data is particularly relevant. Only indicators that can provide data for all countries have been selected and, following the methodology suggested in the *United Nation ESCAP Asia-Pacific Trade and Investment Report*, “if values are available for both an earlier and a later year than the year for which the aggregate is calculated, the missing value has been imputed using linear interpolation. A missing country value for a year preceding the earliest year for which a value is available has been imputed using the value from the earliest year. Similarly, a missing country value for a year following the latest year for which a value is available has been imputed by using the value of the latest year […]. No information is used from other countries for imputing the missing values” (Mikic 2009, p. 182). It is worth remembering that with the purpose to lessen the “risk that European social governance is reduced to mostly technical work programs, unachievable benchmarks, ineffective ‘targetology’” (Cantillon 2017), the indicators that have been used for the computation of the DTF scores are only an example of the set of indicators that can be used to characterize the benchmark. The use of alternative indicators does not change significantly the final results if those alternative indicators are chosen coherently with the definition of each index within the theoretical benchmark. This topic will be, however, object of future research.

## European Challenges and Policy Directions: An Empirical Analysis

### DTF Scores Analysis: Progress and Challenges of European Member Countries

We have considered the period from 2007 to 2017. This period is relevant because the first years precede the European sovereign debt crisis and contains the introduction of the ES. We have considered all 28 European member countries, including UK, which exited after 2017. To grasp the role of institutional variety, we have grouped member countries according to their institutional framework, following the Varieties of Capitalism (VoC) theory approach. VoC theory nowadays goes beyond the traditional classification of Hall and Soskice (2001) that identifies the Liberal Anglo-Saxon Market Economies (LMEs—the Netherlands, UK and Ireland) and the Continental/coordinated Market Economies (CMEs—Austria, Belgium, France, Germany and Luxembourg). Indeed, some scholars (e.g. Dilli et al. 2018) underline the importance to consider the presence of Mediterranean Market Economies (MMEs—Greece, Italy, Portugal and Spain, with the islands Cyprus and Malta) and Eastern European Market Economies (EMEs—post-communist countries such as Bulgaria, Croatia, Czech Republic, Hungary, Poland, Romania, Slovak Republic, Slovenia, Estonia, Latvia and Lithuania). It is useful to also distinguish the countries of Northern Europe that follow a social democratic market (Scandinavian) model of capitalism (SDMEs—Denmark, Finland and Sweden) (see for example Vallejo-Peña and Giachi 2018).

**Fig. 1:**
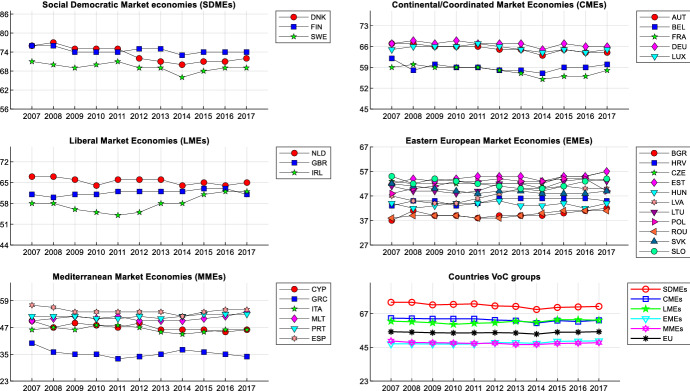
DTF scores for each year (average all indices)

Figure 1 reports the DTF scores from 2007 to 2017 for all European member countries (see in the Supplementary Material Table A for countries’ codes and Table C for the data), considering the average for all indices.

At the outbreak of the subprime mortgage crisis in 2008, the distance from the frontier was already quite significant for EU and little has changed over time. The Scandinavian member countries (i.e. Nordic member countries, including Finland) are on average the closest to the frontier. MMEs and EMEs were quite far from the frontier already in 2007. The improvement in some EMEs has somewhat compensated for the deterioration of most Nordic and continental countries (excluding on average LME countries, see Table C in the Supplementary Material). This seems consistent with the widespread opinion that European funds have contributed to the development of Eastern countries. As expected, Greece is the country that deteriorated most from 2007. It seems that the EU was experiencing political and socio-economic inefficiencies well before the crisis.

**Fig. 2:**
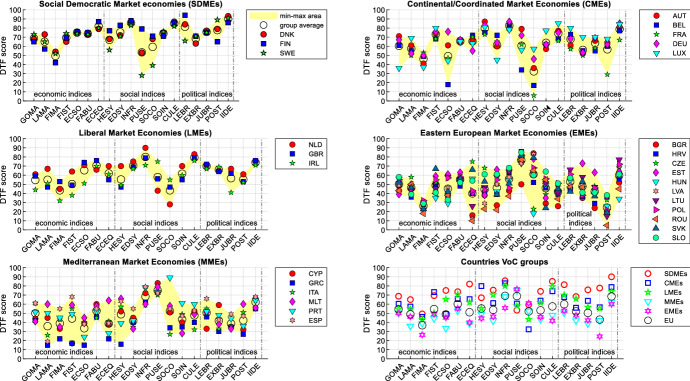
DTF scores for each index and country (average all years)

Figure 2 allows to deepen the analysis of indices’ DTF scores, by considering the average values of the DTF scores for the whole period (see in the Supplementary Material Table B for indices’ codes and Table D for the data).

Despite the peculiarities of the member countries, grouping them according to the varieties of capitalism makes it possible to note that countries that share similar institutional frameworks tend to share rather similar strengths and weaknesses. A partial exception are MMEs and EMEs, where we find a greater internal variety of performances, as the coloured areas highlight. This is also an effect of the use of the geometric mean which penalizes countries with unbalanced profiles (see in the Supplementary Material Figure E and Table F for a comparison with the scores computed using the arithmetic aggregation). Beyond this, however, the use of the arithmetic or geometric mean does not significantly change the conclusions regarding indices analysis and country comparisons. Financial markets and social conflicts are on average the weakest indices for the SDMEs, CMEs and LMEs. For these countries, the strengths are political equality for SDMEs and infrastructure for CMEs and LMEs. On the other hand, public security is a strength of MMEs and EMEs which, however, show on average rather lower scores for other indices such as financial markets, economic equity, economic soundness and labour markets (for MMEs) and political stability (for EMEs). Looking at the European average, the most problematic indices (DTF scores below 50) are financial markets, political stability, labour markets and economic soundness.

**Fig. 3:**
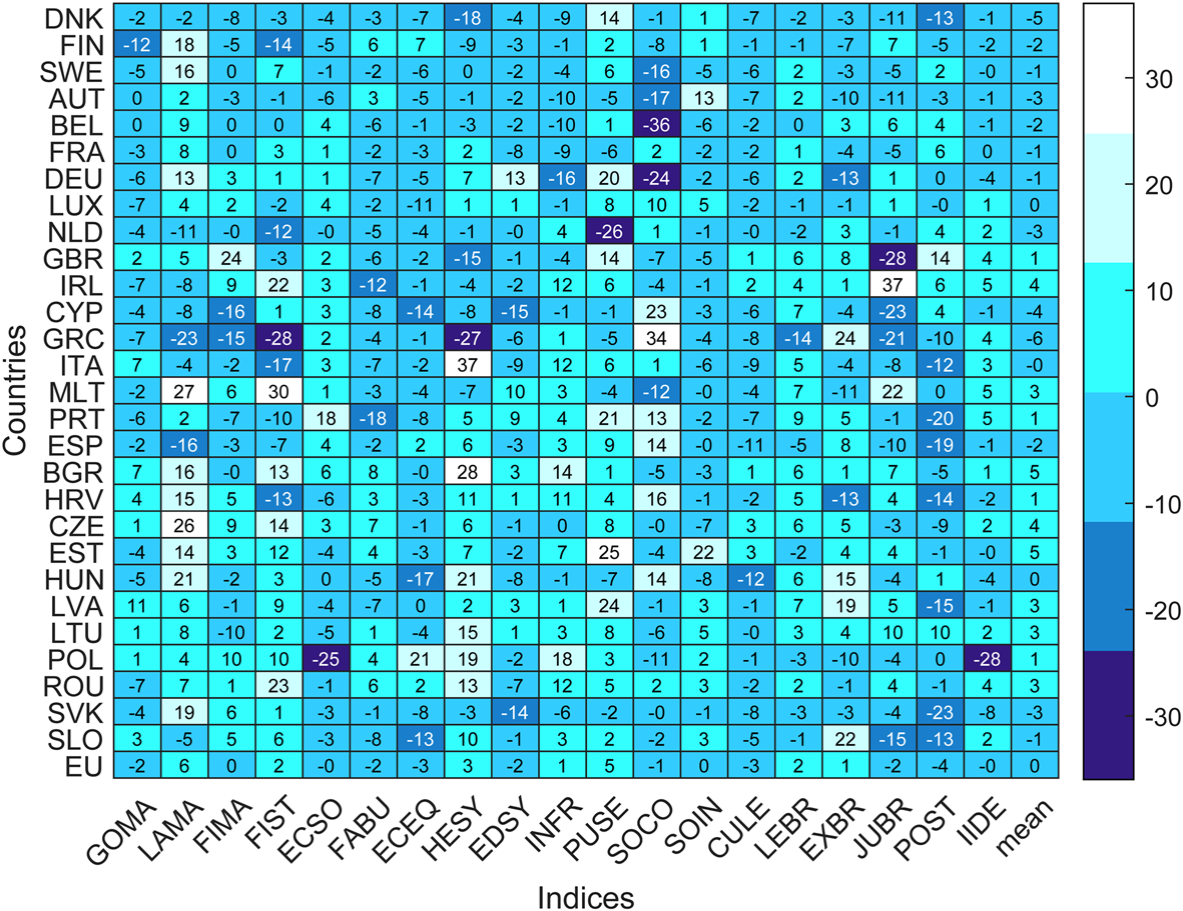
Heatmap of indices dynamics (DTF scores variations from 2007 to 2017)

The heatmap in Figure 3 allows to investigate DTF scores variations from 2007 to 2017. The indices that deteriorated most since 2007 at the European level are political stability (– 4), economic equity and culture of legality (– 3). The impression of a widespread worsening of social equity conditions within member countries is confirmed by other authors (e.g. Sangiovanni 2019). Ten indices have deteriorated since 2007. Fourteen member countries have experienced on average a deterioration of their indices since 2007. From 2007, indices deteriorated on average among Nordic member economies and CMEs, except Luxembourg. Considering all countries and indices, 51% of DTF scores have worsened since 2007. In particular, 22% of scores have lost more than 5 points since 2007.

### A content Analysis of CSRs: A Comparison with DTF Scores

Through the DTF score methodology, we have an idea of the progress made by member countries towards European objectives, considering the economic, social and political perspective. An interesting aspect is to assess the impact of the ES and its CSRs on these results. To do this, we made a comparison between the DTF scores and the more than 1300 CSRs issued from 2011 to 2018 and collected in the CSRs database^2^. First, we tried to classify each CSR according to the 19 benchmark’s indices, where possible. Since each CSR can deal with several topics, we have taken into consideration the more than 2100 sub-CSRs, coherently with other researches on the ES (e.g. Crespy and Vanheuverzwijn 2017; D’Erman et al. 2019; Efstathiou and Wolff 2018; Haas et al. 2020). As noted by D’Erman et al. (2019, p. 199), “CSR texts are highly condensed and technical and even small changes in their formulation can change the meaning drastically”; consequently, often the content analysis is carried out through a manual coding, or by a team of coders (e.g. D’Erman et al. 2019; Haas et al. 2020; Vesan et al. 2021). Our strategy consisted in a search of keywords within the sub-assessment of implementation provided by the EC for each sub-CSR, which are less condensed and more detailed with respect to the original sub-CSRs and reduce (but do not eliminate) the risk of misinterpretations of the policy directions of sub-CSRs, which is what we need for proceeding to our classification. The use of keywords for classifying CSRs and carry on a descriptive content analysis has been already used in other researches on ES (e.g. Clemens and Azzopardi-Muscat 2019; Baeten and Vanhercke 2017). With the aim of reducing discretion in the keyword selection process, we have identified them through a preliminary content analysis (word cloud and bag-of-words analysis) by relying on a software (MATLAB Text Analytics Toolbox). We have selected words suitable to be keywords in the matching procedure between sub-CSRs and benchmark’s indices (see in the Supplementary Material Table J also for more details about the methodology followed in the content analysis). The keywords suitability depends on their coherence with the indices definition as reported in chapter 3.

**Fig. 4:**
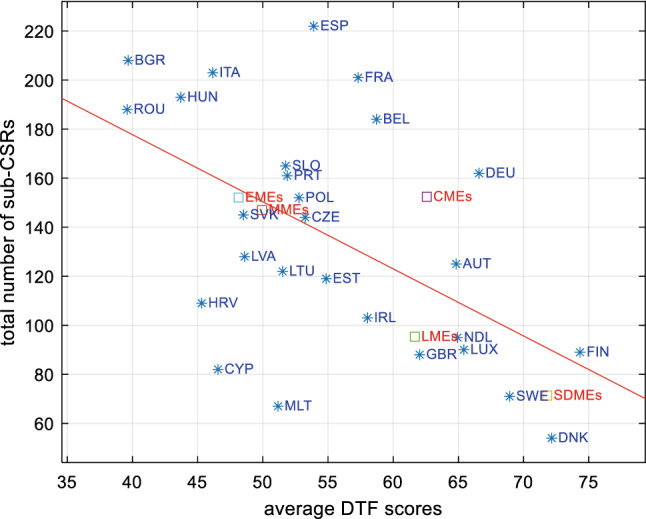
Total number of sub-CSRs and average DTF scores (average all years and indices) of member countries

**Fig. 5:**
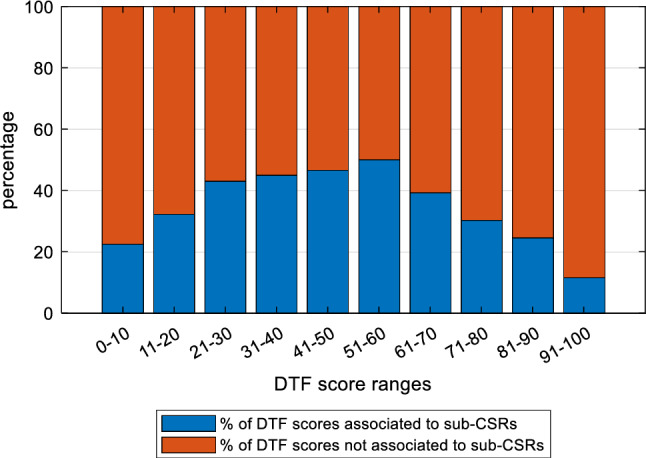
Percentage of DTF scores associated to sub-CSRs (for DTF score ranges) of all countries

The identification of the keywords allows to associate each sub-CSRs to one or more (or none) of the benchmark’s indices. This procedure allows to fragment the sub-CSRs that present more themes, and to consider the complexity of each sub-CSRs so that at the end we come to 3410 associations between sub-CSRs and indices. The sub-CSRs that are not assigned to any index are subjected to a new content analysis procedure aimed at identifying any keyword that had not been previously selected and which would have allowed a matching of the sub-CSRs. This procedure was repeated until the remaining keywords were completely unrelated to the benchmark. These remaining keywords allowed us to assign sub-CSRs to the residual index “other”. This reiteration process allowed us to reduce errors within the matching procedure and to collect useful information on the themes covered by the CSRs but not considered in our benchmark. The total number of associations between sub-CSRs and indices is considered as the effective total number of sub-CSRs to be used in the analysis. Since each sub-CSR has been issued to a member country in a particular year, our classification of sub-CSRs according to benchmark’s indices allowed us to compare the average DTF scores of each country (considering the average of all indices and all years) with the total number of sub-CSRs received by each country and to check whether countries with low DTF scores have received more CSRs, as it is plausible to expect. In Fig. 4, results show that countries that on average perform better have received fewer sub-CSRs (see the red trend line). However, CMEs have received more CSRs compared to other countries and in particular LMEs and SDMEs. In Fig. 5, we report, for ranges of DTF scores, the percentage of scores that has received at least one sub-CSRs, considering all countries and years. Although in general fewer sub-CSRs are associated with better score intervals, a negative relationship is evident for scores above 50.

**Fig. 6:**
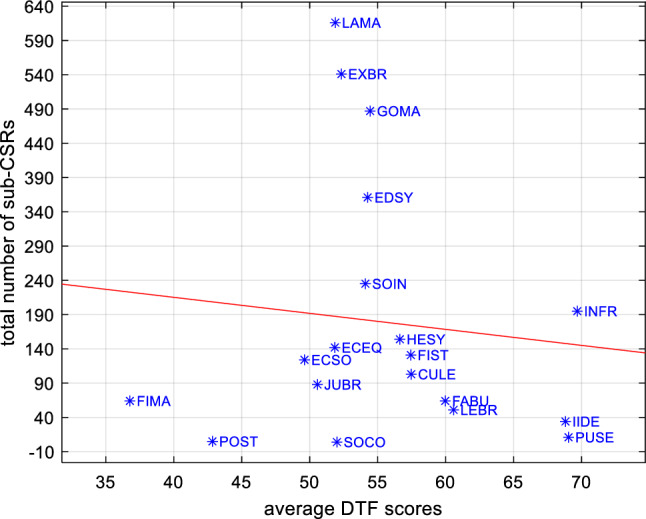
Total number of sub-CSRs and average DTF scores (average all years and countries) of indices

If we consider the relation between the total number of sub-CSRs and the average DTF scores of the indices (average all years and countries) as in Fig. 6, the negative relationship between higher DTF scores and number of sub-CSRs remains valid (see the red trend line) but weaker with respect to Fig. 4.

There is a strong and significant negative correlation between average DTF scores of member countries and number of sub-CSRs received (correlation coefficient equal to − 0.5358 and *p* value 0.0040) but a weak and not significant negative correlation between average DTF scores of the indices and number of sub-CSRs received (correlation coefficient equal to − 0.1038 and *p* value 0.6723). As reported in Table 2 and for each country, by investigating the correlation between the number of sub-CSRs received and the DTF scores for each index from 2011 to 2018, it is possible to note that the correlation is strong and significant (*p* value < 0.05) only for 5 member countries and for one of these countries, surprisingly, the correlation is positive (see bold numbers in Table 2).

**Table 2:** Correlations between number of sub-CSRs and DTF scores for each index, country and year

	DNK	FIN	SWE	AUT	BEL	FRA	DEU	LUX	NLD	GBR	IRL	CYP	ITA	MLT
Correlation coefficient	− 0.03	− 0.14	0.05	− 0.03	0.14	0.05	− 0.12	− 0.05	0.06	− 0.13	− 0.13	− 0.09	− **0.27**	− **0.18**
*p* value	0.76	0.08	0.52	0.72	0.08	0.56	0.14	0.53	0.46	0.11	0.11	0.26	**0.00**	**0.03**

	PRT	ESP	BGR	HRV	CZE	EST	HUN	LVA	LTU	POL	ROU	SVK	SLO
Correlation coefficient	− 0.07	− **0.28**	− 0.13	− **0.17**	− 0.04	0.00	**0.18**	− 0.04	− 0.05	− 0.10	− 0.04	− 0.14	− 0.15
*p* value	0.42	**0.00**	0.12	**0.04**	0.66	0.99	**0.03**	0.59	0.54	0.23	0.62	0.09	0.06

These results suggest that although the number of sub-CSRs is coherent with the average DTF score for most countries, only for a minority of these countries these sub-CSRs have been issued punctually for the indexes that needed more intervention according to DTF scores (see in the Supplementary Material in figure G the DTF scores and the number of sub-CSRs for each country, each index and each year). To investigate this phenomenon, it is necessary to analyse more in depth the composition and content of the sub-CSRs.

### Grasping the Policy Direction of the European Semester and Understanding the Roots of European Deterioration

**Fig. 7:**
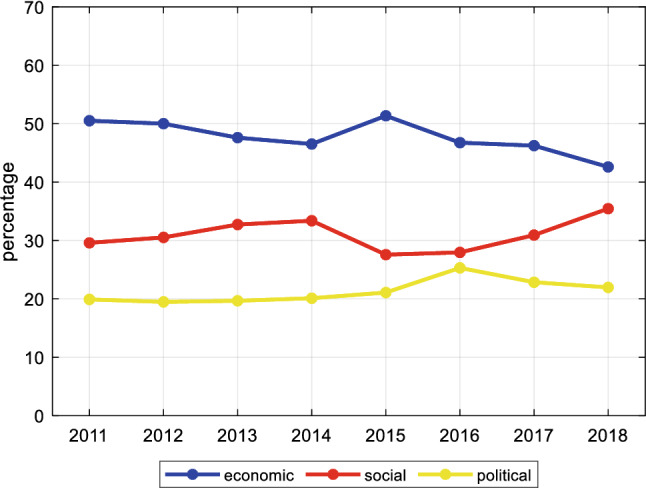
Percentage of sub-CSRs issued for each dimension from 2011 to 2018 (average all countries)

Economic, social and political themes have been always present within sub-CSRs with an increment of the social dimension from 2011 to 2018, although the economic dimension remains predominant (Fig. 7).

**Fig. 8:**
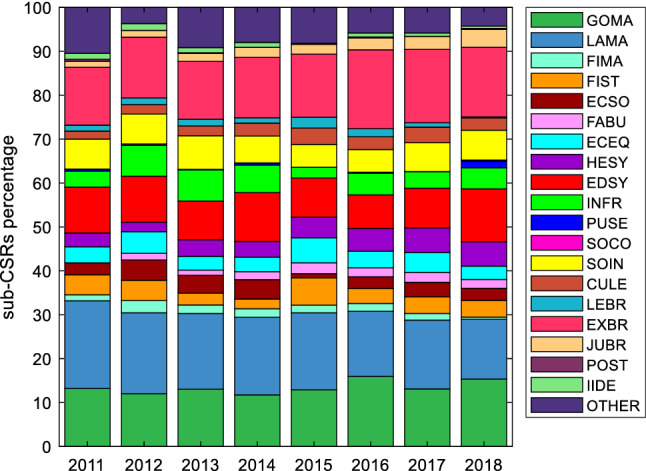
Percentage of sub-CSRs issued for each index from 2011 to 2018 (average all countries)

It is interesting to note a sort of “indices ranking” that seems persistent in time (Fig. 8). Indeed, the efficiency and effectiveness of goods, labour market and the public administration (executive branch) are of major concern among sub-CSRs.

It is surprising the small role of efficiency and effectiveness of financial markets. Some indices, such as public security, social conflict, political stability and equality, play little role within sub-CSRs. Only public security started to be more relevant in 2018.

**Fig. 9:**
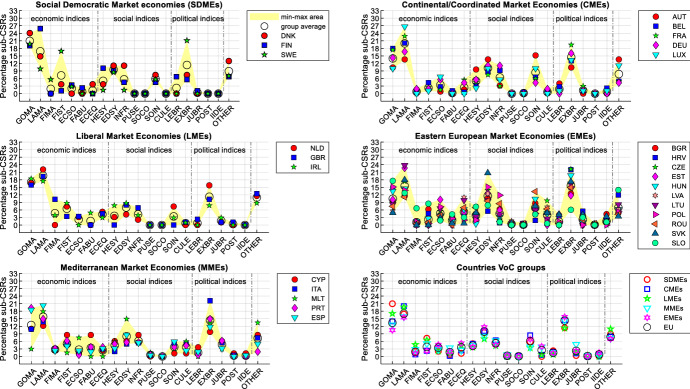
Percentage of sub-CSRs issued for each index for each country

In Fig. 9, the percentages of sub-CSRs issued to each country for each index are reported. For each country, the sum of the percentages of the 20 indices (“other” included) is equal to 100%. Countries have been grouped according to their variety of capitalism to compare Fig. 9 with Fig. 2. Although DTF score analysis suggested that countries with similar institutional frameworks share similar issues and these issues can be different for different varieties of capitalism (Fig. 2), the sub-CSRs seems to identify broadly the same priorities for all varieties of capitalism, despite differences in the number of sub-CSRs issued to single countries (Fig. 9). As discussed previously, only for some countries these priorities match closely the issues that emerge according to the DTF score analysis.

These results may suggest that the principal purpose of CSRs is to promote particular types of structural reforms, whose aim is to reach specific policy objectives valid for all institutional frameworks. This is evident by observing the relevant number of CSRs devoted to improve goods market, labour market and government efficiency and effectiveness, although these indices, on average and according to DTF scores, are not so critical for performance as other indices such as the efficiency and effectiveness of financial markets and their poor ability to allocate resourced in the real economy. This result seems to confirm the findings of Crespy and Vanheuverzwijn (2017, p. 92) who claimed, on the basis of an analysis of all ES documents since 2011, that “despite floating meaning, the notion of structural reforms exhibits a persisting core consisting of typically neoliberal policy recipes such as the liberalization of products and services markets, the deregulation of labour markets, and public administration reform”.

It is interesting to note how the 57% of the sub-CSRs classified exclusively in the first index (efficiency and effectiveness of goods market) deals explicitly with privatizations, liberalizations, promotion of competition and removal of barriers. Moreover, 16% of the sub-CSRs classified exclusively in the second index (labour market efficiency and effectiveness) deal with the promotion of a flexible market in order to reduce labour costs while another 26% deals with the sustainability of pension systems and the need to discourage retirement. An additional 28% of sub-CSRs deal with labour reforms whose impact for workers’ rights is unclear. As noted by Clauwaert (2013, p. 15) “the fact that, over the years, the number of CSRs in relation to ‘adjusting employment protection legislation’ has fallen significantly only indicates that member states have indeed implemented reforms in this area, but tells us nothing about the negative impact of these deregulatory reforms on workers’ individual and collective fundamental social rights”. Similarly, D’Erman et al. (2019, p. 206) ask: “in its recommendation focused on labour markets and wages, does the EU promote reducing or strengthening workers’ rights?”.

Data support the hypothesis that other CSRs within the social dimension may be subordinated to, or a consequence of the pursuit of economic objectives. Table 3 gives an idea of the relations between indices. In each cell is reported the number of sub-CSRs associated both to the index in the row and the one in the column. The sum of the diagonal cells gives the total number of associations between indices and CSRs (3410), while the red colour is associated to high values.

**Table 3:**
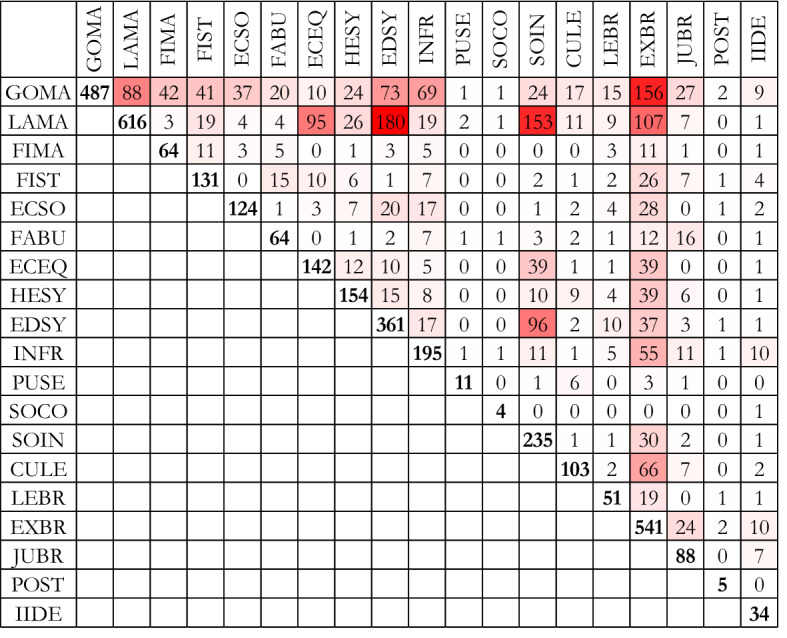
Connections between indices: number of sub-CSRs associated with both the row index and the column index

Goods and labour markets and public administration (executive branch) are involved in many sub-CSRs and indices. In particular, education and social inclusion are strongly connected to the efficiency and effectiveness of markets. Within social inclusion, the most relevant theme is gender and equal opportunities between women and men (46%), disadvantaged and vulnerable groups (46%) and migrants and refugees (8%). Full involvement of women, migrants and disadvantaged groups in the labour market represent the 25% of the sub-CSRs that deal with labour market. Although the aim to involve these groups in labour market is laudable, there are good reasons to suspect that the real aim of these sub-CSRs is to face the problem of ageing population and the sustainability of the pension system by employing the maximum number of people. Also the 8% of sub-CSRs within labour market index devoted to childcare may be interpreted in this sense. Indeed, the aim to contain the costs arising from ageing population is a recurrent theme within sub-CSRs and made explicit within the sub-assessments of implementation provided by the EC. It is repeatedly recommended to discourage retirement and to promote the employment of older workers, and this is done more frequently than recommendations aimed at preventing youth unemployment.

Another relevant (and strictly connected) topic is related to the need to provide labour markets with a professional and flexible workforce. In some sub-CSRs, it is explicitly stated that low-skilled population is a challenge in a context of ageing population and can hinder inclusion, employability and competitiveness. De la Porte and Heins (2015) observe how European institutions aim at “ensuring workers are skilled and adaptable to the altering aims on the labour market” (p. 20) while Hastings and Heyes (2018) note how “greater labour market and contractual flexibility is needed if employers are to meet new competitive challenges, but in return workers should be provided with forms of support, such as access to benefits and lifelong learning, that will enable them to make successful transitions between jobs or between unemployment and employment” (pp. 458–459). Another time, the real purpose of social objectives such as lifelong learning and education is firstly to meet the needs of competitive and flexible markets.

It could be argued that the pursuit of social inclusion, education and economic progress are not conflicting objectives. However, problems in ensuring social and employment protection within a framework in which the primary aim is to reduce labour costs in a context of slowing investment, promote liberalization and competitiveness and guarantee the sustainability of the pension system cannot be neglected. The difficulty in ensuring flexicurity (i.e. policies aimed at ensuring employment growth, social inclusion and workers’ protection) within EU especially after the crisis (Hastings and Heyes 2018) seems to confirm that these concerns are well founded. In addition to this, a lack of cultural integration or cases of exploitation (as can be frequent with migrants) are not considered within sub-CSRs, in spite of the fact that the deterioration of social cohesion and stability testified by the DTF scores may indicate that similar issues are relevant within member countries. Words such as “culture” (also within the education sphere), “social cohesion”, “exploitation” or “workers’ rights” never appear in the sub-CSRs. Although 57% of the sub-CSRs in the economic equity index deals with minimum income and wage, the main topic of these sub-CSRs remains the need to reduce labour costs, review the indexation mechanism and ensure that changes in minimum wage are consistent with job creation and competitiveness. Another time, the impact for workers’ welfare and rights is controversial.

The content analysis of the residual item “other” (see Fig. 8) confirms the great importance for EU institutions to guarantee economic sustainability even regardless of other conditions. Within the “other” category we considered sub-CSRs that have not been classified in any other index. This category represented in 2011 the 10% of all the sub-CSRs, although it declined to 4% in 2018. However, the legal basis of the 76% of these sub-CSRs is the SGP and/or the MIP. On average, the 35% of these CSRs deals with fiscal discipline without a clear reference to the quality and efficiency of public expenditure (which is present in the economic soundness index) and 23% deals exclusively with the sustainability of the pension system and the pressure for discouraging early retirement and connecting retirement age to life expectancy. An additional 2% deals with structural reforms and privatization processes non-clearly connected to any index, while the residual 40% of these CSRs are those that cannot be inserted in any category.

The importance of sustainability and cost-efficiency is recurrent in many indices. Within the index about the efficiency and effectiveness of the health system, 51% of the sub-CSRs deals with the sustainability of the healthcare system and the need to reduce its costs. In this regard, Azzopardi-Muscat et al. (2015) claim that “health systems are not merely a burden on public finances for Europe [...] the fact that all the CSRs for pensions and health are captured under the heading of sustainability of public finances and not that of employment and social policies leads to the conclusion that the debate at European level remains skewed” (p. 381).

In these conditions, also other indices dealing with the social dimension are weakened and subordinated to the needs of fiscal sustainability and cost-efficiency. As confirmed by Haas et al. (2020, p. 336), “some countries receive a mix of recommendations that appears challenging to implement […] the EU often recommends more social protection but also lower spending, which can be problematic as an increase in social protection rarely comes for free. […] Implementing such costly reforms, while also limiting deficits, requires governments to cut spending on other areas, triggering the resistance of affected stakeholders”. In general, Costamagna (2013, p. 16) notes how “most of the recommendations touching upon the functioning of social protection systems or labour market regulation have been strongly concerned with ensuring their financial sustainability and their efficiency, paying limited attention to the effects on their capacity to perform core social functions”.

The “commodification” of all the sub-CSRs reveals a utilitarian and individualistic vision of society that points more to economic performance and fiscal sustainability than to human development. The absence of the word “democracy” within sub-CSRs and the disinterest for the degree of social approval of the EU policy direction, testified by sub-assessments of implementation complaining about the lack of political will to implement controversial reforms, do not help to understand to what extent the CSRs are based on dialogue and shared objectives. As confirmed by Costamagna (2013, p. 24): “such a one-sided approach raises doubts as to its compatibility with a number of Treaty provisions, which impose to EU institutions to find a balance between the pursuit of economic objectives and the safe guard of the European social dimension”.

In general, the observation of Hartlapp (2019, p. 61) seems to be valid when she states that “EU social policy has substantially changed, strengthening its market-supporting dimension, while social policy in its own right has been weakened”. According to our analysis, it is hard to deny that “social Europe is now more strongly arranged to promote market competitiveness and recommodification of labor” (p. 1359) and that “social policy is a function for the common market” (van Gerven and Ossewaarde 2018, p. 1357).

The analysis of the situation of Nordic member countries, whose DTF scores declined since 2007, may be instructive for explaining the roots of the sociopolitical deterioration in terms of stability and cohesion and the role of European strategies in this dynamic. Indeed, these countries have long faced important challenges such as “international integration, demographic changes and changing socio-economic conditions” (Andersen 2004, p. 744). As noted by Andersen et al. (2007), “there are no easy solutions for maintaining a large redistributive welfare state in an environment of ageing populations and intensified global competition” (p. 26). Other authors argue that immigration, considered by many scholars a valid solution to the ageing population problem, seems to represent a problematic issue in the Nordic context. Sanandaji (2015, p. 59) claims that “through immigration Nordic countries have become less homogeneous and thus more unequal” and that Scandinavian socio-economic success “is not immediately translated to migrants” (p. 87). In addition to this “due not least to the redistributive welfare state, immigration from non-western countries to Denmark has so far not been advantageous to the native Danish population” (Nannestad 2004, p. 766) and similar issues can be extended to the other Nordic member countries. In front of a difficult cultural as well as economic integration, it is not surprising that “anti-immigration parties have had considerable success in Scandinavian countries recently” (Sanandaji 2015, p. 96). We cannot exclude that similar problems are also affecting other European countries which indeed show deficiencies in social stability and cohesion.

The excessive subordination of social policies to the objectives of financial sustainability and fiscal discipline, as sustained by many scholars, can only worsen the ability of European countries to face challenges related to ageing population, global competition and integration. Indeed, these challenges require an effort to ensure social equity, inclusion and satisfactory living conditions for all, an accessible and efficient education and health system, and working conditions and perspectives compatible with workers’ rights. Without paying attention to these aspects, sociopolitical cohesion and stability cannot be guaranteed, and integration itself may be challenged.

## Conclusions

The ES and CSRs have been introduced with the purpose to reconcile and promote economic integration and social progress. In order to assess the implementation of this purpose in front of European institutional variety and challenges, we used the distance to frontier (DTF) score methodology. We measured the distance of each member country from a benchmark based on EU aims, values and socio-economic goals defined in the EU treaties. Following the spirit and the letter of the treaties, we considered equally important the economic, political and social dimensions.

According to the results obtained, the EU is quite far from the benchmark and has experienced since 2007 a deterioration of stability and cohesion from an economic, political and social perspective. Surprisingly, this deterioration seems to regard also most Northern countries. A content analysis of the CSRs issued from 2011 to 2018 and a comparison with the DTF scores reveals a weak connection between member countries’ performance and CSRs. This result suggests that the purpose of CSRs is not (or at least not mainly) to solve economic, social and political issues of each member country, but to promote particular types of structural reforms, whose aim is to reach specific policy objectives valid for all member countries.

Despite the social content of many CSRs, we observe a “commodification” of their goals. CSRs promote a society functional to flexible and competitive markets, and compatible with the requirements of fiscal discipline and sustainability. Many issues related to social conflicts, integration and equity issues are not really addressed. This neoliberal approach, quite far from the aims and values of European treatises, may have played a role in the EU deterioration and in the inability to reach the frontier for many European countries facing challenges related to ageing population, global competition and integration. Further research is needed on these issues in order to assess the potential ambiguous implication for European citizens of the policy direction of the ES.

## Supplementary Information

Below is the link to the electronic supplementary material.

Supplementary file1 (DOCX 678 kb)

## Footnotes

1 The used databases are: World Bank, World Economic Forum, International Monetary Fund, UNESCO, OECD, Eurostat, European Commission, Council of Europe European Commission for the efficiency of justice (CEPEJ), Eurofound, Freedom House, Global Financial Development Database, International Institute for Democracy and Electoral Assistance (International IDEA), International Labour Organization (ILO), Quality of Government (QoG) Institute, Sustainable Governance Indicators (SGI), and other sources.

2 The Economic Governance Support Unit (EGOV) publishes an excel database on CSRs, which provides annual information for each member country on CSRs, their legal basis, implementation level and implementation score (www.europarl.europa.eu/cmsdata/211386/CSR%20database_v78_final.xlsm).
