# Supplementary material for: Socio-Economic and Political Challenges of EU Member Countries: Grasping the Policy Direction of the European Semester
Source: Comp Econ Stud. 2021 Oct 11;64(3):487–519. doi: 10.1057/s41294-021-00171-2 (PMC8503392; doi:10.1057/s41294-021-00171-2)
Supplement: Supplementary file 1 — Supplementary file1 (DOCX 678 kb) [file 41294_2021_171_MOESM1_ESM.docx]

**Supplementary Material**

**Table A and B - countries and indices**

**Table C - DTF scores for each year (average all indices)**

**Table D - DTF scores (average all years) of the indices**

**Figure E – DTF scores (average all years) using arithmetic aggregation**

**Table F - DTF scores (average all years) of indices using arithmetic aggregation**

**Figure G – DTF scores and number of sub-CSRs**

**Table H – Source data codes**

| BvD | Bankscope and Orbis Bank Focus, Bureau van Dijk |
| --- | --- |
| CEPEJ | Council of Europe European Commission for the Efficiency of Justice |
| DB | Doing Business |
| EC | European Commission |
| EF | Eurofound |
| EOS | Executive Opinion Survey |
| EUROSTAT | Statistical Office of the European Union |
| FAS | Financial Access Survey |
| FH | Freedom House |
| GCI | Global Competitiveness Index |
| GFDD | Global Financial Development Database |
| GWP | Gallup World Poll |
| IDEA | International Institute for Democracy and Electoral Assistance |
| IFS | International Financial Statistics |
| ILO | International Labor Organization |
| IMF | International Monetary Fund |
| ITU | International Telecommunication Union |
| OECD | Organization for Economic Cooperation and Development |
| QoG | Quality of Government Institute |
| S&P | Global Stock Markets Factbook and supplemental S&P data, Standard & Poor's |
| SAFE | European Central Bank Survey on the access to finance of enterprises |
| SGI | Sustainable Governance Indicators |
| SILC | Statistics on Income, Social Inclusion, and Living Conditions |
| UNESCO | United Nations Educational, Scientific and Cultural Organization |
| WB | World Bank |
| WDI | World Development Indicators |
| WEF | World Economic Forum |
| WEOD | World Economic Outlook Database |
| WFE | World Federation of Exchanges |

**Table I – Indicators**

| **Index** | **Indicator name** | **Sources and Dataset** |
| --- | --- | --- |
| **1.GOMA** | Intensity of local competition | GCI, WEF, EOS |
|  | Extent of market dominance | GCI, WEF, EOS |
|  | Local supplier quantity | GCI, WEF, EOS |
|  | Circular material use rate | Eurostat |
|  | Generation of waste | Eurostat |
|  | Renewable Energy | SGI, WB |
|  | CO2 emissions metric tons per capita | WDI, WB |
|  | Local supplier quality | GCI, WEF, EOS |
|  | Production process sophistication | GCI, WEF, EOS |
|  | Firm-level technology absorption | GCI, WEF, EOS |
|  | Degree of customer orientation | GCI, WEF, EOS |
| **2.LAMA** | Unemployment Rate | ILO |
|  | Labour underutilization | ILO |
|  | Median age of the labour force | ILO |
|  | Labour dependency ratio | ILO |
|  | Older Employment | SGI, Eurostat |
|  | Unemployment rates of young people | Eurostat |
|  | Employment rates of recent graduates | Eurostat |
|  | Horizontal skills mismatch rate | Eurostat |
|  | Vertical skills mismatch rate | Eurostat |
|  | Risk job loss | Eurostat, EF |
|  | Cooperation in labour-employer relations | GCI, WEF, EOS |
|  | Extent of staff training | GCI, WEF, EOS |
| **3. FIMA** | Stock market capitalization to GDP % | GFDD, WFE, S&P |
|  | Stock market total value traded to GDP % | GFDD, WFE, S&P |
|  | Number of ATMs per 100000 adults | GFDD, FAS, IMF |
|  | Number of commercial bank branches per 100000 adults | GFDD, FAS, IMF |
|  | Affordability of financial services | GCI, WEF, EOS |
|  | Availability of financial services | GCI, WEF, EOS |
|  | % of firms that find interest rates or price too high as the most important limiting factor to get financing | EC, SAFE |
|  | % of firms that find financing not available at all as the most important limiting factor to get financing | EC, SAFE |
|  | Lerner index | GFDD, BvD |
|  | Boone indicator | GFDD, BvD |
|  | Financial services meeting business needs | GCI, WEF, EOS |
|  | Financing through local equity market | GCI, WEF, EOS |
|  | Ease of access to loans | GCI, WEF, EOS |
|  | Venture capital availability | GCI, WEF, EOS |
|  | Domestic credit provided by financial sector % of GDP | IMF, IFS, WB |
|  | Private credit by deposit money banks to GDP % | GFDD, IFS, IMF |
|  | Private credit by deposit money banks and other financial institutions to GDP % | GFDD, IFS, IMF |
|  | Firm pressing problem: access to finance | EC/SAFE |
|  | Bank loans: % of firms that did not apply because of possible rejection | EC/SAFE |
|  | Bank loans: % of firms that applied but refused because cost too high | EC/SAFE |
|  | Bank loans: % of firms that applied but was rejected | EC/SAFE |
| **4. FIST** | Stock price volatility | GFDD |
|  | Stabilizing Global Financial Markets | SGI |
|  | Soundness of banks | GCI, WEF, EOS |
|  | Bank Z-score | GFDD, BvD |
|  | Bank nonperforming loans to total gross loans % | IMF |
|  | Gross national savings % GDP | GCI, IMF, WEOD |
|  | Inflation, annual % change | GCI, IMF, WEOD |
| **5. ECSO** | Wastefulness/Efficiency of government spending | GCI, WEF, EOS |
|  | Economic Policy | SGI |
|  | RIA Application | SGI |
|  | Quality of RIA Process | SGI |
|  | Sustainability Check | SGI |
|  | Prevalence of foreign ownership | GCI, WEF, EOS |
|  | Control of international distribution | GCI, WEF, EOS |
|  | Value chain breadth | GCI, WEF, EOS |
|  | R&I Policy | SGI |
|  | Environmental Policy | SGI |
| **6. FABU** | Strength of auditing and reporting standards | GCI, WEF, EOS |
|  | Protection of minority shareholders’ interests | GCI, WEF, EOS |
|  | Efficacy of corporate boards | GCI, WEF, EOS |
|  | Regulation of securities exchanges | GCI, WEF, EOS |
|  | Strength of investor protection | GCI, WB, DB |
|  | Property rights | GCI, ITU |
|  | Legal rights index | GCI, WB, DB |
| **7.ECEQ** | Gini coefficient | Eurostat |
|  | Poverty Rate | SGI |
|  | Pay and productivity | GCI, WEF, EOS |
|  | In work at-risk-of-poverty rate | Eurostat |
|  | Low Pay Incidence | SGI, Eurostat |
|  | Impact of social transfers on poverty reduction | EU-SILC, OECD |
|  | Living conditions (house) | EU-SILC, Eurostat |
|  | Severe material deprivation rate | Eurostat |
|  | Redistribution Effect | SGI, Eurostat |
| **8. HESY** | Medical technology facilities | Eurostat |
|  | Available beds in hospitals | Eurostat |
|  | Self-reported unmet needs for medical examination Reason declared: too expensive | Eurostat |
|  | Self-reported unmet needs for medical examination. Reason declared: too far to travel | Eurostat |
|  | Self-reported unmet needs for medical examination. Reason declared: waiting list | Eurostat |
|  | Cost of seeing the doctor | EF |
|  | Delay in getting appointment to doctor | EF |
|  | Distance to doctor’s office | EF |
|  | Infant mortality, deaths/1,000 live births | GCI |
|  | Deaths related to infectious diseases | Eurostat |
|  | Amenable and preventable deaths of residents | Eurostat |
|  | Quality of health services | EF |
|  | Health Policy | SGI |
| **9. EDSY** | Internet access in schools | GCI, WEF, EOS |
|  | Primary education enrolment, net % | GCI |
|  | Secondary education enrolment, gross % | GCI, WEF, EOS |
|  | Tertiary education enrolment, gross % | GCI, UNESCO |
|  | Availability of research and training services | GCI, WEF, EOS |
|  | Quality of the education system | GCI, UNESCO |
|  | Quality of management schools | GCI, WEF, EOS |
|  | Quality of math and science education | GCI, UNESCO |
|  | Quality of scientific research institutions | GCI, WEF, EOS |
|  | Upper Secondary Attainment | SGI, Eurostat |
|  | Tertiary Attainment | SGI, Eurostat |
|  | PISA results | SGI, OECD |
|  | Availability of scientists and engineers | GCI, WEF, EOS |
|  | University-industry collaboration in R&D | GCI, WEF, EOS |
|  | NEET rates | Eurostat |
|  | Education Policy | SGI |
| **10. INFR** | Quality of roads | GCI, WEF, EOS |
|  | Quality of port infrastructure | GCI, WEF, EOS |
|  | Quality of air transport infrastructure | GCI, WEF, EOS |
|  | Quality of electricity supply | GCI, WEF, EOS |
| **11. PUSE** | Reliability of police services | GCI, WEF, EOS |
|  | Safe Living Conditions | SGI |
|  | Population reporting occurrence of crime, violence or vandalism in their area | Eurostat |
|  | Recorded crimes police data: Intentional or attempted homicide | Eurostat |
|  | Recorded crimes police data: Assault and kidnapping | Eurostat |
|  | Recorded crimes police data: Sexual violence, rape or sexual assault | Eurostat |
|  | Recorded crimes police data: Robbery | Eurostat |
|  | Recorded crimes police data: Burglary and theft | Eurostat |
|  | Recorded crimes police data: Unlawful acts involving controlled drugs or precursors | Eurostat |
|  | Business costs of terrorism | GCI, WEF, EOS |
|  | Business costs of crime and violence | GCI, WEF, EOS |
| **12. SOCO** | Perceived tension between different racial and ethnic groups | EF |
|  | Perceived tension between different religious groups | EF |
|  | Perceived tension between poor and rich people | EF |
|  | Attended a protest or demonstration | EF |
| **13. SOIN** | Gender employment gap | OECD |
|  | Gender pay gap | Eurostat |
|  | Low-skilled Unemployment | SGI |
|  | Social exclusion index | EF |
|  | Social Inclusion Policy | SGI |
|  | Integration Policy | SGI |
| **14. CULE** | Irregular payments and bribes | GCI, WEF, EOS |
|  | Tax gap | Raczkowski (2015)* |
|  | Diversion of public funds | GCI, WEF, EOS |
|  | Organized crime | GCI, WEF, EOS |
|  | Corruption Prevention | SGI |
|  | Shadow economy | Raczkowski (2015)* |
|  | Ethical behaviour of firms | GCI, WEF, EOS |
|  | Favouritism in decisions of government officials | GCI, WEF, EOS |
|  | Safeguards against official corruption | FH |
| **15. LEBR** | GO Expertise | SGI |
|  | GO Gatekeeping | SGI |
|  | Line Ministries | SGI |
|  | Cabinet Committees | SGI |
|  | Ministerial Bureaucracy | SGI |
|  | Government Efficiency | SGI |
|  | Self-monitoring | SGI |
|  | Parliamentary Resources | SGI |
|  | Obtaining Documents | SGI |
|  | Summoning Ministers | SGI |
|  | Summoning Experts | SGI |
|  | Task Area Congruence | SGI |
|  | Audit Office | SGI |
|  | Ombuds Office | SGI |
|  | Transparency of government policymaking | GCI, WEF, EOS |
|  | Government openness and transparency | FH |
|  | Confidence in national government | GWP, WB, WDI |
|  | Trust in the government | EF |
| **16. EXBR** | Tax System Complexity | SGIWB DB |
|  | Burden of government regulation | GCI, WEF, EOS |
|  | Burden of customs procedures | GCI, WEF, EOS |
|  | Business impact of rules on FDI | GCI, WEF, EOS |
|  | No procedures to start a business | GCI, WB, DB |
|  | No days to start a business | GCI, WB, DB |
|  | Cost of business start-up procedures % of GNI per capita | WB, DB |
|  | Firm pressing problem: regulation | EC, SAFE |
|  | Effect of taxation on incentives to invest | GCI, WEF, EOS |
|  | Effect of taxation on incentives to work | GCI, WEF, EOS |
|  | Tax Policy | SGI |
| **17. JUBR** | Efficiency of legal framework in settling disputes | GCI, WEF, EOS |
|  | Efficiency of legal framework in challenging regs | GCI, WEF, EOS |
|  | Clearance rate | CEPEJ |
|  | Disposition time | CEPEJ |
|  | Judicial Review | SG |
|  | Trust in the legal system | EF |
| **18. POST** | Number of changes in government per year | QoG |
|  | Political participation | EF |
|  | Electoral Self-Determination | QoG |
|  | Voter Turnout | IDEA |
|  | Voting-age population VAP turnout | IDEA |
|  | Public trust in politicians | GCI, WEF, EOS |
| **19. IIDE** | Fairness of the electoral laws and framework | FH |
|  | Independence of the elected head of government and national legislative representatives | FH |
|  | Judicial independence | GCI, WEF, EOS |
|  | Judiciary independence | FH |
|  | Appointment of Justices | SGI |
|  | Intra-party Democracy | SGI |
|  | Legal Certainty | SGI |
|  | Negotiating Public Support | SGI |

* Raczkowski, K. 2015. Measuring the tax gap in the European economy. Journal of Economics and Management 21(3), 58-72.

**Table J - Keywords and content analysis methodology**

| **index** | **keywords codes** |
| --- | --- |
| **GOMA** | busines*, business service, service provider, compan*, professional service, services sector, enterpr*, industr*, business digitalisation, craftsmen, craft, e-commerce, SMES, housing, state-owned enterpris*, SOE , SOEs, professional certificate, business environment, industrial park, corporate sector, entrepreneur, entrepreneurship, industrial activit*, market unity, grocery store, building sector, construction sector, farmer, postal market, rental market, rent*, rental, competition authority, retail*, tourism, barriers competition, competition restrictions, competition service sector, regulated profession, regulated professions, regulation profession, deregulation profession, dominant market position |
| **LAMA** | unemployment, employment, wage*, worker, job*, employee, hire, hiring, jobseeker, career, salar*, recruitment, traineeship, workforce, jobless, precarious, workbonus, flexjob, labour market, open-ended contract, apprenticeship, collective bargaining, wage bargaining, wage setting, childcare, unemployed, public servant, second earner, employability, labour instrument, work capacity, working capacity, collective agreements, ALMPS, professional pathway, reskilling, parental leave system, labour code, disincentives work, labour relations |
| **FIMA** | access finance, access venture capital, microcredit, microfinance, functioning business bank, finance business, financing SMEs, loan, corporate governance bank, competition banking sector, lending, access credit, state-owned bank, finance corporate, banks corporate governance, nationalised bank, banking sector action plan, consolidation banking sector, landesbanken sector, savings bank, reforms banking, financial instruments, bank asset amangement |
| **FIST** | banking supervision, securitisation, deleverage, leveraging, non-performing, undercapitalised, mortgage, illiquid, NPL, asset quality, financial stability, stress test, bad asset, bad loan, households debt, early warning, non-resident banking, impaired asset, credit risk monitoring, liability risk, portfolio cleaning, recapitalisation, liquidity, capital ratio, concentration risk, related-party exposure, recapitalised, bank funding stress, supervision of bank, resilience of the banking sector, debt overhang, supervision bank, high indebtedness, deleveraging, capitalised, inflation, bad bank, banks’ workout capacity, financial supervisory authority, household debt, macroprudential authority, supervisory framework financial sector, loan resolution |
| **ECSO** | quality public spending, foreign exposure, predictability decision-making, regulatory impact assessment, strategic planning, streamlining priorities, long-term vision, quality predictability policies, innovation polic*, energy policy, promotion renewable energy, high-quality social service, support innovative, research development, supervision cross border entities, climate policy, diversifying energy sources, economy measures adopted foster innovation, efficiency public investment, efficiency public spending, energy efficiency, energy generation efficiency, growth-enhancing expenditure, growth-friendly, improve growth prospects, social protection system, prioritising growth, promote energy renewable sources, public expenditure efficiency, public expenditure research innovation, public investment project, public research, public-sector reform, regulate prices, sound fiscal position, state research agency, support public investment, targeting social policies measures, top sectors business strategy, transparency public finances, national research polic*, national innovation strategy |
| **FABU** | consumer protection, property rights, transparency tariff setting mechanism, transparency wage, title deed, disclosure, insolvency, pre-insolvency, credit regist*, late payments, payment delay, contract enforcement, consumer authority |
| **ECEQ** | poverty, social justice, anti-poverty, equitable, low-income, low-income earner, minimum income, inequality, social benefit, minimum inclusion income, social transfer, social assistance benefit, income guarantee, minimum wage, social assistance, cash benefit, sickness benefit, social security coverage |
| **HESY** | health, healthcare, hospital, patient, medicinal, medicine, ambulatory, medical, doctor, pharmac*, bed*, cancer, clinical, e-health, cholesterol, oncology, disease, policlinics, long-term care |
| **EDSY** | education, school*, researcher*, vocational, learning, teach, professional qualifications student, university*, science, academia, graduate, enrolment, academic, mathematics, pedagogical, polytechnic, scholarship, scientific, classroom, interuniversit*, lower secondary, maths, PISA, scientist, NEETs, bachelor, research institution, attainment basic skills |
| **INFR** | infrastructure, transport, electricity, gas, telecommunication, railway, broadband, electrification, tunnel, hydroelectric, electromobility, water, pipeline, airport, electric, rail service, port*, ports, motorway, energy system |
| **PUSE** | criminal, crimes, police, illegal, illegally |
| **SOCO** | tense climate, improving social dialogue, social dialogue law |
| **SOIN** | disadvantaged, inclusion, inclusive, inclusiveness, women, migrant, disabilit*, migration, emigration, gender, social exclusion, refugee, discriminatory, segregat*, solidarity, asylum, low-skilled, Roma, underrepresented group, immigrant, discrimination, vulnerable group, PES, youth guarantee, equal opportunit*, rights child |
| **CULE** | shadow economy, undeclared work, tax avoidance, corruption, anticorruption, conflict interest, tax evasion, fraud, favouritism, ethic, informal payment, lobbying, absenteeism, antifraud, transparency public procurement |
| **LEBR** | legislative framework, layers government, lack coordination administration, lack coordination various authorit*, institutional coordination, late approval, government reform, delays legislation, lack administrative capacity, reform local, municipal structure, interconnection capacity, implementation gap, overlapping responsibilit*, strengthening analytical capacity key ministr*, coordination among different levels government |
| **EXBR** | permit*, e-invoice system, tax administration, fiscal framework, tax system, administrative procedure, administrative simplification, public administration, public tender, parafiscal, tax, bureaucracy, administrative, taxation, red tape, administrative burden, burden labour, e-government, e-governance, zoning, e-invoic*, building licence, public procurement, VAT, regulatory burden, state administration, procurement procedure, ease start-up, regulatory environment |
| **JUBR** | civil justice, digitalisation civil trial, court, magistracy, civil procedural rule, justice system, court proceeding, pending case, length court proceeding, judicial, justice, judiciary, judge, lawyer, legal service, civil procedure, judicial, litigation |
| **POST** | more stable parliamentary majorit*, political instability, electoral law, political opposition, legislative instability |
| **IIDE** | independence, monitoring responsibilit*, transparency judicial system, independent body, political cycle |

CSRs database has been imported from excel to MATLAB using the Text Analytics Toolbox, which provides algorithms for pre-processing and analysing text data (<https://it.mathworks.com/help/textanalytics/>). The sub-CSRs have been imported as strings. The raw text has been cleaned for analysis by removing punctuation, symbols, noise, upper cases and stop words. Strings have been tokenized so that to transform them into collections of words. Starting from a word cloud and bag-of-words analysis, the keywords in Table D have been selected. The algorithm that made it possible to perform the association of the sub-CSRs with the indices took into account the various declinations of the keywords (e.g., plural forms, upper cases, presence of symbols etc.). A similar methodology has been followed for the analysis of TEU and the TECE.
